# Extending Local Canonical Correlation Analysis to Handle General Linear Contrasts for fMRI Data

**DOI:** 10.1155/2012/574971

**Published:** 2012-01-23

**Authors:** Mingwu Jin, Rajesh Nandy, Tim Curran, Dietmar Cordes

**Affiliations:** ^1^Department of Physics, University of Texas at Arlington, Arlington, TX 76019, USA; ^2^Department of Radiology, School of Medicine, University of Colorado Denver, Aurora, CO 80045, USA; ^3^Departments of Biostatistics and Psychology, UCLA, Los Angeles, CA 90095, USA; ^4^Department of Psychology and Neuroscience, University of Colorado at Boulder, Boulder, CO 80309, USA

## Abstract

Local canonical correlation analysis (CCA) is a multivariate method that has been proposed to more accurately determine activation patterns in fMRI data. In its conventional formulation, CCA has several drawbacks that limit its usefulness in fMRI. A major drawback is that, unlike the general linear model (GLM), a test of general linear contrasts of the temporal regressors has not been incorporated into the CCA formalism. To overcome this drawback, a novel directional test statistic was derived using the equivalence of multivariate multiple regression (MVMR) and CCA. This extension will allow CCA to be used for inference of general linear contrasts in more complicated fMRI designs without reparameterization of the design matrix and without reestimating the CCA solutions for each particular contrast of interest. With the proper constraints on the spatial coefficients of CCA, this test statistic can yield a more powerful test on the inference of evoked brain regional activations from noisy fMRI data than the conventional *t*-test in the GLM. The quantitative results from simulated and pseudoreal data and activation maps from fMRI data were used to demonstrate the advantage of this novel test statistic.

## 1. Introduction

The General Linear Model (GLM) is a widely used mass univariate analysis method to determine brain activations in functional magnetic resonance imaging (fMRI) because of its simplicity in both estimation and inference and its greater sensitivity to regional effects than global multivariate analyses [1]. The least-squares (LS) solution of the GLM is the minimum variance unbiased (MVU) estimator when Gaussian white noise assumption is satisfied, otherwise the weighted LS solution (using the inverse of the noise covariance matrix) becomes the best linear unbiased estimator (BLUE) [2]. The estimated parameters and their variances are used to construct various contrast statistics, either *t* or *F*, to test the null hypothesis of effects of interest. Another popular approach to analyze fMRI time series uses the correlation coefficient [3]. The statistical significance of the correlation coefficient is equivalent to a *t*-statistic testing for a regression on one single regressor [4]. The correlation coefficient is more restricted in assessing the significance of regional effects than the *t*-test in fMRI data analysis because the correlation coefficient does not allow more than one regressor to be included for a direct calculation. It is known, however, that the partial correlation coefficient is also equivalent to a *t*-test and thus could potentially be used instead. However, each contrast of interest need be constructed and the residuals, after removing effects of no interest, have to be calculated for each contrast. This process is generally less computationally efficient than the *t*-test used in the GLM.

While univariate (single voxel) analysis is extensively applied in fMRI, and temporal correlations are the focus of most investigations, only a few applications investigate the spatial dependence of fMRI data. Univariate analysis deals only with a uniform nonlocal spatial approach and uses fixed isotropic spatial Gaussian smoothing routinely to achieve more homogeneous regions of activation and to control the family-wise error parametrically, based on the theory of random fields [5, 6]. These methods do not utilize local spatial information in fMRI data, and fixed spatial smoothing causes unnecessary blurring of the edges of activation. More severely, if the fixed isotropic filter kernel is larger than the activated area, it could potentially miss the detection of activated regions. Small focal regions of low contrast-to-noise ratios are rather common in episodic memory paradigms where the task is to detect activation in the medial temporal lobes (hippocampus and parahippocampus). Therefore, fixed Gaussian spatial smoothing can potentially result in missing important (but subtle) focal activations. This is especially troublesome for high-resolution fMRI data where the intrinsic point spread function of the imaging sequence is not much larger than the dimension of a voxel and there are sharp boundaries between grey matter and surrounding cerebrospinal fluid (CSF) and blood vessels (see, e.g., [7]).

A more effective method than fixed Gaussian spatial smoothing uses locally adaptive spatial filter kernels. Using the spatial dependence of fMRI data, local multivariate methods such as canonical correlation analysis (CCA) [8] and its variants [9–13] have the ability to significantly increase the detection power of fMRI activations. However, there are several drawbacks that prevent CCA methods from being widely used in fMRI analysis. First, the original unconstrained CCA method [8] increases the number of false positives due to more freedom in finding favorable linear combinations with nonactive voxel time series leading to a decrease in specificity. This drawback can be addressed by either enforcing some constraints on the spatial coefficients [10, 12, 13] or adaptively assigning the canonical correlation to the most significant voxel [11]. Second, these modified CCA methods [10, 11, 13] usually require much more computation time than the GLM and the unconstrained CCA method. Jin et al. [12] proposed a region-growing strategy to solve the constrained CCA (cCCA) problem in a much faster fashion than the traditional branch-and-bound method [10, 13, 14]. Third, in the form of previous implementations, CCA applications in fMRI data analysis were very limited because test statistics used were based on the significance of the maximum canonical correlation coefficient, thus limiting the analysis to a simple model accommodating only one temporal regressor (i.e., on-off experimental design). This drawback prevents researchers from using CCA for more complicated paradigms with multiple explanatory variables and nuisance covariates in fMRI. Though reparameterization based on the linear contrast of interest can provide a solution for this drawback [15, 24], the computational cost is high because, for each different reparameterization, the constrained CCA problem needs to be solved. The major goal of this research is to find a suitable test statistic for CCA that allows the testing of general linear contrasts and that is also fast.

In this paper, we first establish the connection between the multivariate multiple-regression (MVMR) model and CCA. Although this is not totally new in statistics, we found that there is lack of awareness for the development of CCA methods in the fMRI data analysis community. By treating the estimated spatial filter kernel of constrained CCA as a linear transformation of the original MVMR model, we further derive a novel univariate test statistic similar to a *t*-statistic based on general hypothesis tests of the MVMR model. This extension will allow CCA to be used for inference of general linear contrasts in more complicated fMRI designs without solving the constrained CCA problem for each particular contrast of interest.

In the following, we start from the MVMR model and its hypothesis test for general linear contrasts under a linear transformation of the original model. Then, the simultaneous estimation of spatial and temporal parameters using the LS rule in the MVMR model is derived and proved to be the same as the CCA solution. By treating the adaptive spatial smoothing as a linear transformation of the original MVMR model, a novel directional statistic for CCA similar to a *t*-statistic can be derived to allow for testing of general linear contrasts. Using receiver operating characteristic (ROC) techniques [16–18] on pseudoreal fMRI data [11, 19–21], we quantitatively compare the sensitivity and specificity of the proposed novel CCA statistic with the *t*-statistic of the GLM without and with fixed Gaussian spatial smoothing. We also apply a nonparametric approach [22] to estimate the family-wise error rate for all methods using resampled resting-state data and show the activation maps for real fMRI data for a simple visual cortex activation paradigm and also for a more complicated memory paradigm.

## 2. Theory

### 2.1. The MVMR Model

Considering a group of *K* local neighborhood voxels, the MVMR model can be written as

(1) Y=XB+E,

where  **X** is fixed (i.e., the *n* × *p* design matrix), **Y** = (**y**_1_, **y**_2_,…, **y**_*K*_) is the matrix containing *K* neighboring voxels,  **B** = (**β**_1_, **β**_2_,…, **β**_*K*_) is the parameter matrix to be estimated, and **E** = (**ε**_1_, **ε**_2_,…, **ε**_*K*_) is the error matrix. Without of loss of generality, **X** and **Y** are column centered and there is no constant column in **X**. When the error matrix satisfies (i) *E*(**E**) = 0, (ii) cov⁡(**ε**_*i*_) = *𝚺*  for *i* = 1,…, *n*, and (iii) cov⁡(**ε**_*i*_, **ε**_*j*_) = 0  for *i* ≠ *j*, the LS solution of the model (1) is equivalent to the BLUE, which is just the matrix form of the univariate GLM estimator leading to equivalent solutions, but a multivariate test need be adopted. Note that conditions (i)–(iii) may not be true for fMRI data, but may be reasonably satisfied using temporal whitening.

The hypothesis tests in the MVMR model can be conducted using the error matrix and the hypothesis matrix for any estimable general linear contrast matrix **C**′. For a linear transformation of the original MVMR model, say **M**, Wilks' Λ and other test statistics (e.g., Roy's largest root) can be used for testing the null hypothesis **C**′**B** **M** = **O** [23]. In addition to the fixed linear transformation of the MVMR model, we will introduce estimation of the spatial filter kernel (leading to an adaptive smoothing) and treat it as a spatially variable linear transformation in the following development. This linear transformation can be estimated from the data using CCA. Utilizing the spatial and temporal coefficients from CCA and the hypothesis test on the linear transformation of the MVMR model, a directional (one-sided) statistical test for CCA can be derived that is similar to a *t* statistic in the GLM. This novel statistic allows CCA to test hypothesis on general linear contrasts of an fMRI design without reparameterization of the design matrix and without reestimation of the CCA solutions for each particular contrast of interest.

### 2.2. Adaptive Filtering through Canonical Correlation Analysis (CCA)

To increase detection power of weak activations, local spatial filtering is usually applied to decrease the noise variance. Let **α** be the vector containing the spatial filtering coefficients, then multiplication of both sides of the MVMR model of (1) with **α** gives

(2) Yα=Xβ+ε,

where **β** ≡ **B** **α**, **ε** ≡ **E** **α**, and multiplication by **α** defines a linear transformation of the original MVMR model in (1).

When both **Y** and **α** are fixed and treated as known, such as in conventional fixed Gaussian smoothing, **β** can be easily estimated by linear regression as

(3) $$\tilde{\beta }={\left(X^{\prime }X\right)}^{-1}X^{\prime }Y\alpha .$$

Given a general linear contrast **C**′, the null hypothesis of **C**′**B** **α** = **C**′**β** = 0 can be tested using Wilks' Λ likelihood ratio test (assuming independent identical normal distribution of noise both spatially and temporally) by

(4) $$\Lambda =\frac{\left|E\right|}{\left|E+H\right|},$$

where the error matrix is $E={\left(Y\alpha -X\tilde{\beta }\right)}^{\prime }(Y\alpha -X\tilde{\beta })$ and the hypothesis matrix is $H={\left(C^{\prime }\tilde{\beta }\right)}^{\prime }{\left[C^{\prime }{\left(X^{\prime }X\right)}^{-1}C\right]}^{-1}(C^{\prime }\tilde{\beta })$. Note that both matrices reduce to a scalar due to the linear transformation of the original MVMR model by vectors **α**.

A fix-sized and isotropic smoothing kernel, such as a Gaussian kernel, is not optimal, especially for weak and small activations. Our goal is to increase detection power by pooling the neighboring voxels with similar activation pattern and by determining the spatial weights **α** from the data as well. This adaptive smoothing can be achieved by minimizing the square of fitting error (i.e., LS) for the model in (2), which leads to the equivalent solution in CCA.

Assuming that the optimal configuration of **Y** is known (please see [10, 12] for how to find this configuration), the vectors **α** and **β** can be estimated by LS:

(5) $$\left(\tilde{\alpha },\tilde{\beta }\right)=\arg\underset{\alpha ,\beta }{\min}{||Y\alpha -X\beta ||}^{2}.$$

There is a trivial solution for (5): $\tilde{\alpha }=\tilde{\beta }=0$, which can be avoided by enforcing some normalization condition, such as ${\tilde{\alpha }}^{\prime }S_{yy}\tilde{\alpha }=1$ or ${\tilde{\alpha }}^{\prime }\tilde{\alpha }=1$. Taking the partial derivative of the square of fitting error over **α**, we get

(6) $$\frac{\partial {||Y\alpha -X\beta ||}^{2}}{\partial \alpha }=2\left(Y^{\prime }Y\alpha -Y^{\prime }X\beta \right).$$

The solution $\tilde{\alpha }$ requires (6) equal to zero so that

(7) $$\alpha ={\left(Y^{\prime }Y\right)}^{-1}Y^{\prime }X\beta .$$

Meanwhile, the relationship in (3) is still valid. Therefore, only one vector needs to be estimated and the other can be determined by (3) or (7). Substituting (3) in (7), we get

(8) $$\begin{array}{cc} \alpha & ={\left(Y^{\prime }Y\right)}^{-1}Y^{\prime }X{\left(X^{\prime }X\right)}^{-1}X^{\prime }Y\alpha \\ & =S_{yy}^{-1}S_{yx}S_{xx}^{-1}S_{xy}\alpha , \end{array}$$

where the sample covariance matrices are **S**_*yy*_ = (1/(*n* − 1))**Y**′**Y**, **S**_*xx*_ = (1/(*n* − 1))**X**′**X**, and **S**_*xy*_ = **S**_*yx*_′ = (1/(*n* − 1))**X**′**Y**. This is an eigenvalue problem for **α** with eigenvalue 1, whose solution may not exist because the eigenvalue of **S**_*yy*_^−1^ **S**_*yx*_ **S**_*xx*_^−1^ **S**_*xy*_ is not necessarily identical to 1. Thus, a conventional method to solve (8) is to write it as an LS problem by

(9) $$\tilde{\alpha }=\arg\underset{\alpha }{\min}{||\alpha -S_{yy}^{-1}S_{yx}S_{xx}^{-1}S_{xy}\alpha ||}^{2}.$$

Given that **α** ≠ 0 by enforcing the normalization condition mentioned previously, the expression ||**α**−**S**_*yy*_^−1^ **S**_*yx*_ **S**_*xx*_^−1^ **S**_*xy*_ **α**||^2^ can be minimized if $\tilde{\alpha }$ is the eigenvector of **S**_*yy*_^−1^ **S**_*yx*_ **S**_*xx*_^−1^ **S**_*xy*_ which has the eigenvalue *λ*_*m*_ closest to 1 (or in other words, the largest eigenvalue of **S**_*yy*_^−1^ **S**_*yx*_ **S**_*xx*_^−1^ **S**_*xy*_ because its upper bound is 1), that is,

(10) $$S_{yy}^{-1}S_{yx}S_{xx}^{-1}S_{xy}\tilde{\alpha }=\lambda_{m}\tilde{\alpha }.$$

Equation (10) results in the same solution for CCA, where *λ*_*m*_ = *r* ^2^ and *r* is the maximal canonical correlation. This is not totally unexpected because

(11) $$\begin{array}{cc} \left(\tilde{\alpha },\tilde{\beta }\right) & =\arg\underset{\alpha ,\beta }{\min}{||Y\alpha -X\beta ||}^{2}\\ & =\arg\underset{\alpha ,\beta }{\min}{||\frac{Y\alpha }{\sqrt{\alpha^{\prime }S_{yy}\alpha }}-\frac{X\beta }{\sqrt{\beta^{\prime }S_{xx}\beta }}||}^{2}\\ & =\arg\underset{\alpha ,\beta }{\min}\left(n-1\right)C_{1}\\ & \;+\left(n-1\right)C_{2}-2\left(n-1\right)\frac{\alpha^{\prime }S_{yx}\beta }{\sqrt{\alpha^{\prime }S_{yy}\alpha }\sqrt{\beta^{\prime }S_{xx}\beta }}, \end{array}$$

where *C*_1_ = **α**′**S**_*yy*_ **α** and *C*_2_ = **β**′**S**_*xx*_ **β** are nonzero constants. Therefore, we can use CCA, which maximizes the third term in the above equation, to find solutions for the model in (2). Once $\tilde{\alpha }$ has been determined, the temporal coefficients $\tilde{\beta }$ can be obtained by (3) accordingly.

Normally, we can achieve a desired filtering effect by adding constraints on the components of **α** in a constrained CCA (cCCA) form. In this work, we constrain all components of **α** to have the same sign. This constraint not only enforces a smoothing effect, bus also has an optimal solution through searching CCA solutions of the possible configurations of **Y** in a prescribed local region to satisfy this constraint [10, 12, 14]. In addition we add a center voxel significance constraint by requiring that the spatial weight of the center voxel be at least 20% of the maximum spatial weight in each 3 × 3 neighborhood [12]. Although this introduces some nonlinearity to the optimization [24], in the current implementation, this additional constraint was found empirically to be effective in producing the best performance. A similar approach was used in [10] to increase the spatial specificity.

Generally, we suggest scaling the solution $\tilde{\alpha }$ of cCCA to have a sum of magnitudes to be one. Although this is not required because the scaling factor will be cancelled out in calculating the novel CCA test statistic (refer to (14) in next section), this treatment can keep the error term comparable with GLM methods. A region-growing method [12] allowing a much faster implementation than the traditional branch-and-bound method [10, 14] will be used to obtain $\tilde{\alpha }$.

Several advantages of the current implementation of cCCA over the method proposed in [10] are listed here. (1) In [10], a spatial Gaussian filter was divided into one isotropic central part and three oriented parts. The weights for these parts can be estimated using CCA to achieve anisotropic filtering (steerable spatial filtering). In our method, we search for the optimal voxel combinations and weights in a 3 × 3 neighborhood because the cortical layer in a typical fMRI scan is less than 5 mm and spans only a couple of voxels. Our smaller filter size can help better define activations leading to higher specificity (2) A rather slow branch-and-bound (BB) method was used in [10], which is not efficient to search optimal combinations for the center voxel in a 3 × 3 area (see Section 5). Our region-growing method takes 24 s for a 2D slice with 6317 in-brain pixels and is much faster than the BB method (308 s) [12] (3) The statistic used in [10] was the maximum canonical correlation coefficient, which can only be used for simple on-and-off paradigms but not for arbitrary linear functions (contrasts) in complicated paradigms. The new statistic proposed in our work can be applied for complicated paradigms without reestimating for each contrast of interest. Although it would be an interesting followup to compare different CCA methods, such a comparison is beyond the scope of the current paper.

### 2.3. Novel Directional Test Statistic for CCA

As a simple treatment, the estimated components of $\tilde{\alpha }$ can be used as local spatial filter coefficients to smooth the original data. Then, the same univariate inference as the GLM can be applied to get a statistical map for any general linear contrast. However, this procedure has two drawbacks: (1) the GLM estimation of **β** on the smoothed images adds extra unnecessary computation time; (2) the resulting statistics will be biased because it does not account for the loss of degrees of freedom caused by the size of the spatial filter kernel. For example, a single voxel configuration is more significant than a multiple-voxel configuration having the same value of the test statistic. To overcome these two drawbacks, we derive the test statistic directly from the CCA coefficients $\tilde{\alpha }$ and $\tilde{\beta }$ and account for the spatial kernel size by changing the degrees of freedom.

Given the general linear contrast **C**′, the null hypothesis: $H_{0}:\;C^{\prime }B\tilde{\alpha }=C^{\prime }\tilde{\beta }=0$ can be tested by Wilks' Λ in (4), where **α** in the error matrix is replaced by $\tilde{\alpha }$. In this paper, we are particularly interested in a directional test statistic when the contrast matrix **C**′ reduces to a vector **c**′. Thus, the test statistic on $c^{\prime }\tilde{\beta }$ reduces to a univariate case with a signed value and can be defined by

(12) $$\Lambda_{\pm }=\mathrm{sign}\left(c^{\prime }\tilde{\beta }\right)\Lambda =\mathrm{sign}\left(c^{\prime }\tilde{\beta }\right)\frac{\left|E\right|}{\left|E+H\right|},$$

where Λ_+_ indicates the positive statistic for values $c^{\prime }\tilde{\beta }>0$ and Λ_−_ indicates the negative statistic for values $c^{\prime }\tilde{\beta }<0$.

Going one step further, we can define a statistic *t*_*c*_ bearing a similar form as the conventional *t*-statistic by writing

(13) $$\Lambda_{\pm }=\mathrm{sign}\left(c^{\prime }{\tilde{\beta }}_{c}\right)\frac{1}{1+t_{c}^{2}/\text{DF}},$$

where DF = *n* − *p* − *K* specifies the degrees of freedom (DOF) given that the number of observations is *n*, the number of (nonconstant) regressors is *p* (linear equations for **β**), and the size of voxel configuration in CCA is *K* (constraints for **α**). As we will discuss next, *t*_*c*_ is not a real *t*-statistic, but rather using the concept of DOF to account for the voxel configuration size similar to *t*-statistic. Thus, a non-parametric estimation method [22] is essential to assess its statistical significance. Since the right sides of (12) and (13) are equal, this statistic can be written by using the definition of **E** and **H** as

(14) $$t_{c}=\frac{c^{\prime }\tilde{\beta }\sqrt{\text{DF}}}{\sqrt{c^{\prime }{\left(X^{\prime }X\right)}^{-1}c}\sqrt{{\left(Y\tilde{\alpha }-X\tilde{\beta }\right)}^{\prime }\left(Y\tilde{\alpha }-X\tilde{\beta }\right)}}.$$

Note that the voxel configuration size has been accounted for in (14) so that the same $c^{\prime }\tilde{\beta }$ values with less voxels become more significant. The new statistic reduces to a traditional *t*-statistic for the single voxel (*K* = 1) case (when the noise is white and Gaussian distributed) given by

(15) $$t=\frac{c^{\prime }\tilde{\beta }\sqrt{n-p-1}}{\sqrt{c^{\prime }{\left(X^{\prime }X\right)}^{-1}c}\sqrt{{\left(y-X\tilde{\beta }\right)}^{\prime }\left(y-X\tilde{\beta }\right)}}.$$

Generally, (14) will not follow a *t*-distribution even under the assumption of independent identical normal distribution of noise in both space and time because of the constrained CCA estimation for **α**. Without spatial correlation in the single voxel case (*K* = 1), (15) can approximate fairly well a *t*-distribution when prewhitening is applied to decorrelate the temporal serial correlations. Moreover, the spatial correlation of fMRI data will pose a tricky problem for approximating a true *t*-distribution. To deal with these difficulties, a non-parametric estimation method [22] is adapted to assess the significance of the CCA statistic of (14). The distribution of this novel statistic on null data will be shown to deviate from the true *t*-distribution in Section 4.

From (14), we can see the advantage of the newly developed test statistic. First, if activations exist at the center voxel and its neighbors, we get a more accurate estimate of $\tilde{\beta }$ (as shown in simulations in Section 4) by pooling these voxels in the estimation. Second, the error term ${\left(Y\tilde{\alpha }-X\tilde{\beta }\right)}^{\prime }(Y\tilde{\alpha }-X\tilde{\beta })$ is always smaller than ${\left(y-X\tilde{\beta }\right)}^{\prime }(y-X\tilde{\beta })$. Therefore, no matter what contrast vector **c** is used, *t*_*c*_ has a larger value than the univariate *t*. It would be expected that *t*_*c*_ values of active voxels increase more than *t*_*c*_ values of voxels in the null state, which will lead to an increased sensitivity. Third, the better model fitting by pooling more voxels is penalized by the degrees of freedom DF = *n* − *p* − *K*. This penalty will cause considerable bias when *n* is comparable to *p* + *K*. However, this scenario is not practically meaningful because the length of the fMRI sequence is usually much greater than the sum of the number regressors and the size of the filter kernel (i.e., *n* ≫ *p* + *K*). 

Note that the proposed test statistic may not necessarily be the optimal test for an arbitrary contrast because we only minimize the square of fitting error in (2) that is independent of the contrast [24]. Nevertheless, the new statistic allows us to improve the detection power without reparameterization of the design matrix and without re-estimating each particular contrast of interest as shown in Section 4. 

## 3. Methods

### 3.1. Imaging Data

Functional MRI (fMRI) was performed at the Brain Imaging Center of the University of Colorado Denver in a 3.0T GE HDx MRI scanner equipped with an 8-channel head coil and parallel imaging technology. Stimulus presentation was done with a rear projection system (AVOTEC, Inc.). Two different paradigms (visual paradigm and memory paradigm) were performed on two and eight healthy adult subjects, respectively, and fMRI data were collected according to local IRB approval. The pulse sequence to collect fMRI data was EPI with the following parameters: ASSET = 2, ramp sampling, TR = 2 sec, TE = 30 ms, FA = 70 deg, FOV = 22 cm × 22 cm, slice thickness = 4 mm, gap = 1 mm, 25 slices, and in-plane resolution 96 × 96. For the visual paradigm we prescribed axial slices and collected 150 volumes, whereas for the memory paradigm we prescribed coronal oblique slices perpendicular to the long axis of the hippocampus and collected 288 volumes. The first 5 volumes were discarded to establish signal equilibrium of the imaging sequence.

To obtain an accurate gray matter mask that has equivalent features of the echo-planar data (same geometry and distortions), we collected for each subject an additional coplanar IR-SE-EPI scan to get inverted T1 contrast with the following parameters: TI = 505 ms, ASSET = 2, ramp sampling, TR = 6 sec, TE = 30 ms, FOV = 22 cm × 22 cm, slice thickness = 4 mm, gap = 1 mm, 25 slices, and in-plane resolution 96 × 96. This imaging sequence yields unique high signals for gray matter so that we can easily threshold them to get accurate gray matter masks. The IR-SE-EPI images were first aligned to the mean EPI images using six-parameter affine transformation and then were thresholded to get gray matter masks. Visual inspection of masks for faithfulness was conducted before calculating the activation voxels in gray matter.

Furthermore, we acquired a coplanar standard high-resolution T2-weighted anatomical scan (FOV 22 cm, resolution 256 × 256, TR 3000 ms, TE 85 ms, NEX 2, slice thickness 4 mm, gap 1 mm). The mean EPI functional image of each individual was coregistered to its corresponding T2 image, and the same transformation was applied on all functional images. The resultant activation map shown in Section 4 was overlaid on the individual T2 image.

#### 3.1.1. Visual Paradigm

**Figure 1:**
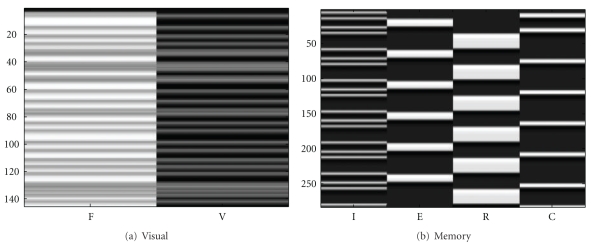
Design matrices for visual (a) and memory (b) paradigms. From left to right, the regressors are fixation (F) and visual activation (V) for the visual paradigm (a), and instruction (I), encoding (E), recognition (R), and control (C) for the memory paradigm (b). The SPM-type two-gamma function was used as the HRF. Note that with centered data, (a) can be modeled by a single centered activation column and (b) can be modeled by three centered columns. The redundant presentation was used to show all experimental conditions.

For each subject we acquired two fMRI data sets. The first data set was collected during resting state where the subject tried to relax and refrained from executing any overt task with eyes closed. The second data set was collected while the subject was looking at a flashing checkerboard (10 Hz flashing frequency, duration 2 sec) which alternated with a fixation period of random duration (2 sec to 10 sec, uniformly distributed). During the fixation period a black screen containing in the center a small white cross (about 1 inch in size) was shown and the subject was instructed to focus on this cross. The corresponding design matrix using the canonical hemodynamic response function (HRF) model is shown in Figure 1(a). The left column in this figure represents the regressor for the fixation and the right column represents the regressor for the visual activation.

#### 3.1.2. Memory Paradigm

Also here, we acquired two fMRI data sets for each subject. The first set contained resting-state data, and the second set was acquired while the subject performed a memory task. Behavioral responses were collected during the memory paradigm with button response pads that the subject had in each hand. The memory paradigm started with a fixation period of 16 sec followed by six identical 89 sec long cycles of “5 sec instruction,” “21 sec encoding,” “5 sec instruction,” “11 sec control,” “5 sec instruction,” and “42 sec recognition”. It ended with another fixation period of 16 sec. The short “instruction period” consisted of a single sentence and reminded the subject of the following task to be performed. The “encoding” task consisted of a series of novel pictures, where each picture was displayed for 3 sec, and the subject was instructed to memorize each picture. During the “control” task the subject saw the letters “Y” or “N” which appeared in random order every 100 ms on the display screen. The subject was instructed to press, as fast as possible, the right button when “Y” appears or the left button when “N” appears. The purpose of the “control” task was twofold. First, it served as a distraction task to keep attention away from the just learned pictures. Second, due to its simplicity it did not produce any activation in regions associated with the memory circuit (hippocampal complex, posterior cingulate cortex, precuneus, and fusiform gyrus). During the “recognition” task the subject saw a series of pictures where half of the pictures were novel and the other half of the pictures were identical to the pictures from the previous “encoding period.” The arrangement of these pictures was random. Each picture was displayed on the screen for 3 sec. The subject was instructed to press the right button if the picture was seen before in the previous encoding period and to press the left button if the picture was identified to be novel and not seen in the previous encoding period. The design matrix using the canonical HRF model is shown in Figure 1(b). The four conditions of “instruction,” “encoding,” “recognition,” and “control” are denoted as “I,” “E,” “R,” and “C,” respectively.

Due to the complexity of the memory paradigm, all subjects were trained on a computer in a quiet room with the paradigm using a different set of images before fMRI scanning. The stimuli presentations were programmed in EPRIME and all button presses were recorded.

### 3.2. Preprocessing

All data were preprocessed in SPM5 using realignment to correct for motion artifacts, slice timing correction to correct for differences in image acquisition time between slices, and high-pass filtering using *T* = 150 sec to remove low-frequency components and signal drifts. The classic two-gamma HRF was used to construct the design matrix. In the next section, we give examples for the contrasts “Visual minus Fixation” (denoted as “V-F”) for visual data and “Encoding minus Control” (denoted as “E-C”) for memory data and ignore other possible contrasts of interest.

### 3.3. Methods of Data Analysis

Three methods were investigated using the statistics defined in (14) and (15). The first two using (15) are (i) the GLM without smoothing, denoted as “GLM-NS” and (ii) Gaussian smoothing followed by the GLM, denoted as “GLM-GS.” The third one is cCCA with the region-growing method [12] using (14), denoted as “cCCA-RG.” The full width at half maximum (FWHM) of Gaussian smoothing in the GLM was chosen as 2.24 pixels. This number is not only falling in the generally recommended smoothing size (2-3 times of the spatial resolution) in fMRI data analysis, but is also equal to the average size of all possible 256 configurations within a 3 × 3 pixel area that includes the center pixel [24].

### 3.4. Construction of Simulated and Pseudoreal Data

In demonstrating the estimation and detection performance of different methods, real fMRI data, where the subject performed a certain paradigm, are difficult to use since the ground truth about the activated regions is unknown. To draw any firm conclusions about the performance of a method, it is better to use simulated/pseudoreal data, where the important parameters are known and can be tested for and the data features (especially the noise characteristics) are similar to real fMRI data [11, 17]. In this work, we always use the resampled resting-state fMRI data as the noise background to preserve the noise characteristics of real data and superimpose either artificial activations or activations extracted from real activation fMRI data. Even though the difference between simulated/pseudoreal data and real data cannot be avoided, the evaluation provides a ranking of the estimation and detection performance of difference methods that is unlikely to change for real data.

To quantitatively determine the performance of different methods, we constructed both simulated and pseudoreal data by defining

(16) $$x=\{\begin{array}{cc} \left(1-f\right)x_{\text{act}}+fx_{\text{null}}, & x\in \text{active}\;\text{set},\\ x_{\text{null}}, & \text{otherwise}. \end{array}$$

In this equation **x** is the vector representing the time series of a voxel with activation contribution **x**_act_ and noise contribution **x**_null_. The noise fraction parameter *f* is a scalar number to adjust the noise level in the data vector **x** given that **x**_act_ and **x**_null_ have the same power. For null data **x**_null_, Fourier resampling [25] of resting-state fMRI data was used to randomize the phase of each time series without destroying the inherent temporal and spatial correlations in the data. Note that there are other resampling methods for fMRI data, such as wavelet resampling [26, 27] and whitening resampling [28–31], and some comparisons have been made based on different criteria [27, 31–33]. Compared to whitening resampling, both Fourier and wavelet resamplings do not assume a specific model (such as AR(p) or ARMA(p,q)) to do model fitting and are thus more general since different voxels may follow different whitening models. To avoid complicating our simulation, we chose Fourier resampling with the same phase permutation for all time series to preserve the spatial correlations of resting-state fMRI data. This resampling method is least computationally demanding and was demonstrated to have a similar ROC performance to that of wavelet resampling [33].

To define different spatial patterns of activations for simulated data, 100000 randomly shaped activations within a 3 × 3 grid of pixels having a size of 2 to 9 pixels were generated. The center pixel was always assigned to be active. The corresponding time courses for the activated pixels **x**_act_ were simulated to be linear combinations of the 4 random temporal regressors with random amplitudes *β*_1_, *β*_2_, *β*_3_, and *β*_4_ uniformly distributed in [0,1]. Different levels of noise introduced by resampled 3 × 3 patches of resting-state fMRI were used for **x**_null_. Both **x**_null_ and **x**_act_ were normalized to have unit variance before the mixture.

To quantitatively evaluate both sensitivity and specificity of the novel CCA test statistic of (14) in comparison with a GLM-based *t*-test in a more realistic setting using ROC techniques [16–18], we constructed pseudoreal data [11] using a combination of activation data and resting-state data. First, GLM-NS was applied on the activation data. Next, the groups of highly active voxels using an unadjusted *P* value threshold of 10^−8^ for the *t*-maps of V-F in visual data and of E-C in memory data were labeled as active, that is, **x**_act_. Finally, we generated, by Fourier resampling of resting-state data, the null data **x**_null_ and constructed the final pseudoreal data according to (16).

**Figure 2:**
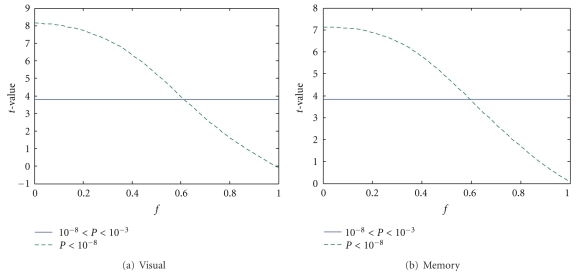
Determination of the proper value for the noise fraction *f* for pseudoreal data. The solid horizontal lines represent the medians of *t*-statistics at the significance level 10^−8^ < *P* < 10^−3^ (uncorrected) for the contrasts V-F (a) and E-C (b) by applying GLM-NS on real fMRI activation data. The dashed curves are the medians of the *t*-statistics of activation-defined voxels for the contrasts V-F (a) and E-C (b) by applying GLM-NS on pseudoreal data where the true activations were defined by thresholding real activation data using a very high significance level (*P* < 10^−8^ uncorrected) and adding resampled noise according to (16) for different noise fractions*f* ∈ [0,1]. The medians of the *t*-value matched at around *f* = 0.6. Therefore, we picked two values for *f* : *f* = 0.55 representing the low noise case and *f* = 0.65 representing the high noise case.

To find the proper noise fraction parameter *f* in (16), we applied GLM-NS on pseudoreal data for *f* ∈ (0,1) with step size 0.01. The median of corresponding *t*-values of activations was compared with the median of *t*-values with significance level in [10^−8^, 10^−3^] by applying GLM-NS on real (non simulated) fMRI activation data. We plotted *t*-values of contrasts V-F and E-C in Figures 2(a) and 2(b), respectively. As can be seen, *f* = 0.6 is a value that two medians match. Therefore, we picked two values for *f* : 0.55 representing the low noise case and 0.65 representing the high noise case. By normalizing the peak variances of noise and signal to be the same, these two values for *f* correspond to a peak signal-to-noise ratio of 67% and 29%, respectively. The logic of choosing these significance levels for determining a proper *f* is the following. Voxels with significance level *P* < 10^−8^ are signals with very high SNR (which are almost certainly true activations), those with significance level in the interval [10^−8^, 10^−3^] are the majority of signals with medium or low SNR and of interests of detection (whose median of the *t*-statistic was used to find a matching *f*), and those with significance level *P* > 10^−3^ are dominated by noise and are therefore ignored.

The advantage of constructing pseudoreal data using real activation data and resampled resting-state data is that the spatial and temporal correlations of both the activations and the noise are similar to real data and the locations of active and nonactive voxels are known by construction. This type of simulation then allows conventional ROC techniques to be applied.

### 3.5. Determination of Proper *P*-Value

To compare different test statistics using real visual and memory activation data, it is necessary to get the proper *P*-values for the corresponding *t*- or novel CCA statistic that is adjusted for multiple comparisons. In this work, we used a non-parametric technique [22]. A non-parametric technique is suitable for a reliable comparison between different analysis methods because the parametric distribution of the CCA statistic is intractable due to the data-adaptive spatial filtering kernel. In the following we outline how the family-wise error rate (FWE) is being calculated using Fourier resampled resting-state data using bootstrapping of the order statistics. For more details, please see the publication [22].

The multiple comparison problem is relevant when we have a family of hypotheses {*H*_*ω*_ : *ω* ∈ *Ω*} at voxel *ω*. Let the test statistics at voxel *ω* be denoted by *Y*_*ω*_. Then FWE is determined by the maximum statistic (max⁡*Y*_*ω*_), and for any threshold *u*, we can calculate the *P-*value that automatically adjusts for multiple comparisons. To estimate the null distribution of {max⁡*Y*_*ω*_}, we use the bootstrap method applied to the *k* largest order statistics {*Y* ^1^,…, *Y* ^*k*^} from Fourier resampled resting-state data. This method is quite general and may be applied to a broad class of test statistics in fMRI. In the present context of CCA, the relevant test statistic is given by (14) or (15). Although it is not strictly necessary, it is preferable to make a transformation of the test statistic using the known (approximate) distribution or the kernel density estimation. We calculate the negative logarithm of the *P-*value corresponding to the test statistic to obtain our transformed variables. Due to the monotonous nature of the transformation, without loss of generality, we can assume that *Y* is already transformed. Define {*d*_*i*_ = *i*(*Y* ^*i*^ − *Y* ^*i*+1^), *i* = 1,…, *k*} as normalized sample spacings for the *k* largest order statistics. If the observed samples at the voxels are exponential i.i.d then so are the normalized sample spacings [34]. This is true since the transformed test statistic is an exponential random variable. The *k* largest order statistics can then be expressed as a linear function of the normalized sample spacings and *Y* ^*k*+1^ as follows:

(17) $$Y^{j}=Y^{k+1}+\overset{k}{\underset{i=j}{\sum }}i^{-1}d_{i},\;j=1,\ldots ,k.$$

Since {*d*_*i*_, *i* = 1,…, *k*} are i.i.d., we can use the bootstrap method to obtain resamples of normalized spacings {*d*_i_*, *i* = 1,…, *k*}. The latter can be used to generate resamples {*Y* ^∗1^,…, *Y* ^∗*k*^} of the *k* largest order statistics from which the distribution of {max⁡*Y*_*ω*_} can be obtained numerically. Since Fourier resampled resting-state data are considered to be null, the obtained distribution can be considered to be the null distribution of {max⁡*Y*_*ω*_}. It can be shown that, under certain regularity conditions, for a suitably chosen *k*, the normalized spacings are i.i.d. asymptotically [35]. Due to the large number of voxels in consideration, the asymptotic result is applicable in the present context. The chosen value for *k* was 100 for the bootstrap method and FWE was computed for *P* = 0.05.

## 4. Results

### 4.1. Estimation of Temporal Coefficients for Simulated Data

We computed the mean square errors (MSE) between the estimated temporal coefficients of the linear combination and the original ones generating the simulated data (Table 1) for a random noise fraction parameter *f* uniformly distributed in [0,1]. The GLM-GS method is inferior to GLM-NS due to the small and irregular defined activations. The cCCA-RG method performs best and has an improvement of more than 25% of MSE in estimating the temporal coefficients. This experiment demonstrates the superior estimation performance of temporal coefficients $\tilde{\beta }$ by the adaptive smoothing capability of cCCA.

### 4.2. Null Distribution of the Proposed Test Statistic

**Figure 3:**
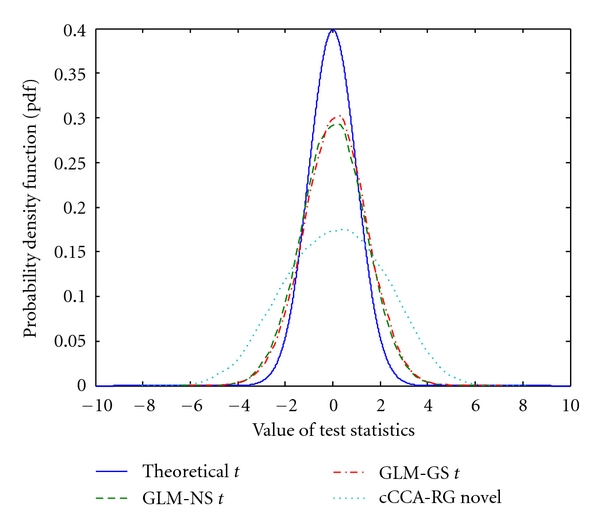
Distributions of the proposed CCA statistic (“cCCA-RG novel”) along with the conventional *t*-statistic used in the GLM using resampled resting-state data and contrast E-C for the memory paradigm. The difference of the GLM-based *t*-statistic from a theoretical *t*-distribution (blue solid curve, DOF = 278) was mainly caused by the temporal correlation in fMRI signal. The novel CCA statistic has the widest profile because of the additional spatial modeling.

Although the proposed novel CCA test statistic has a similar form as the *t*-statistic in the GLM, its null distribution deviates significantly from the theoretical *t*-distribution as we mentioned previously. To shed more light on this issue, we applied different methods using Fourier resampled resting-state data and the contrast vector for the memory paradigm to get the null distributions of the contrast E-C. The results were plotted in Figure 3. The theoretical *t*-distribution with a DOF of 278 is also plotted for reference. It can be seen that all distributions are wider than the theoretical *t*-distribution, even for the GLM methods. Meanwhile, since *n* is much greater than *p* and *K* in this case, the adjustment induced by *K* in (14) is almost negligible. Since the distribution of the novel CCA test statistic has a complicated structure and is difficult to parameterize, it is necessary to use non-parametric methods to determine significance values accurately.

### 4.3. Area under the ROC Curve for Pseudoreal Data

We computed the area under the ROC curve (called “AUR”) for a false positive fraction (FPF) less than 0.1 as an index of detection performance. The AUR quantity provides a weighted measure of detection power for specificities larger than 0.9 (which is the most interested range for fMRI data).

**Figure 4:**
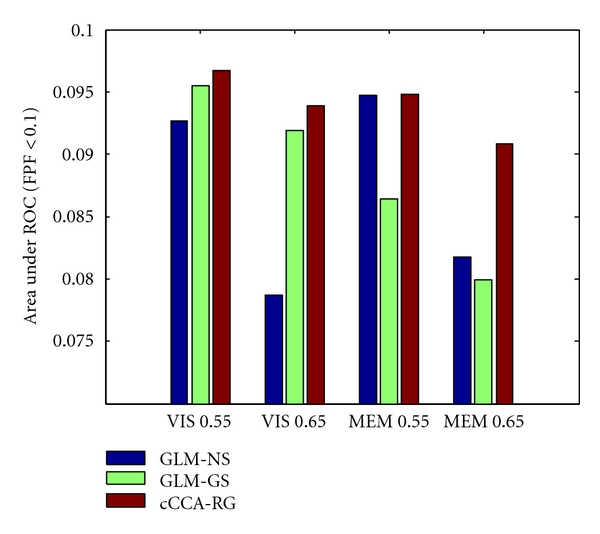
Detection performance of different data analysis methods showing the area under the ROC curve (AUR), integrated over FPF ∈ [0,0.1], using pseudoreal data. The AURs for the contrast V-F of the visual paradigm (“VIS 0.55” and “VIS 0.65”) and the contrast E-C of the memory paradigm (“MEM 0.55” and “MEM 0.65”) are shown for the low noise case (*f* = 0.55) and the high noise case (*f* = 0.65), respectively. The cCCA-RG achieves the greatest AUR values in all cases.

 The AUR quantities for the contrast V-F of the visual data and for the contrast E-C of memory data are shown in Figure 4. Since the induced activations at the visual cortex are spatially extended, Gaussian smoothing (GLM-GS) yields better detection performance than GLM-NS. However, when activations are more irregular in shape and spatially localized as in the memory task, Gaussian smoothing produces adverse effects and GLM-GS consequently performed worse than GLM-NS. As can be seen, cCCA-RG always yields the top performance in all cases. The biggest advantage of cCCA-RG is in detecting small activations from a high noise background (“MEM 0.65”).

**Figure 5:**
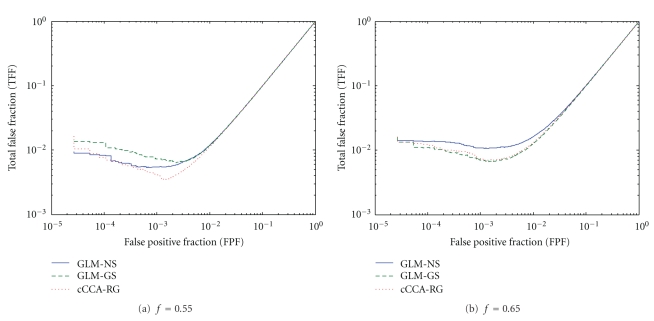
The total false fraction (TFF) (including false positives and false negatives) versus the false positive fraction (FPF) for the contrast V-F of the visual paradigm for pseudoreal data: (a) the low noise case (*f* = 0.55) and (b) the high noise case (*f* = 0.65). Note that all TFF curves have minima in the interval [0.001,0.01]. The cCCA-RG performs nearly optimal in both cases by achieving the minimum value of TFF.

**Figure 6:**
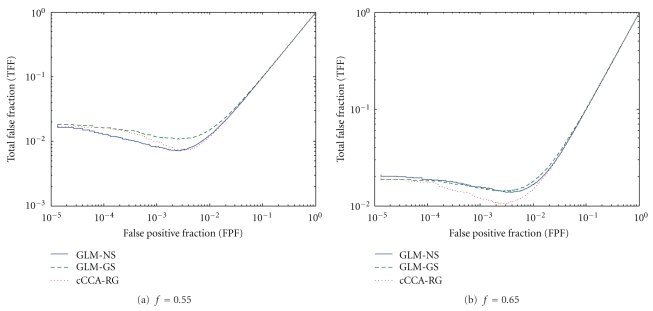
The total false fraction (TFF) (including false positives and false negatives) versus the false positive fraction (FPF) for the contrast E-C of the memory paradigm for pseudoreal data: (a) the low noise case (*f* = 0.55) and (b) the high noise case (*f* = 0.65). Note that all TFF curves have minima in the interval [0.001,0.01]. The cCCA-RG method is optimal in both the low and high noise cases.

 In addition, we plotted the curves for the total false fraction (TFF) (including both false positives and false negatives) versus the false positive fraction (FPF) in Figure 5 (for the contrast V-F of the visual data) and Figure 6 (for the contrast E-C of the memory data). This measurement provides another perspective on the detection performance of different methods. For the extended activations of the contrast V-F, cCCA-RG achieves the smallest TFF at *f* = 0.55 (Figure 4(a)), followed by GLM-NS and GLM-GS. The GLM-GS method is effective in the high noise case (Figure 4(b) *f* = 0.65) and performs similar to cCCA-RG. In Figure 5, for the small activations of the contrast E-C, cCCA-RG remains the optimum and yields much more improved performance over other methods in the high noise case (*f* = 0.65). The GLM-GS method works poorly even in the high noise case. This demonstrates that it is destructive to apply fixed Gaussian spatial smoothing on the data with small activations. Constrained CCA combined with the proposed test statistic is more reliable and thus a better alternative to detect these activations.

### 4.4. Activation Maps Using Real Data (with Corrected *P* < 0.05)

**Figure 7:**
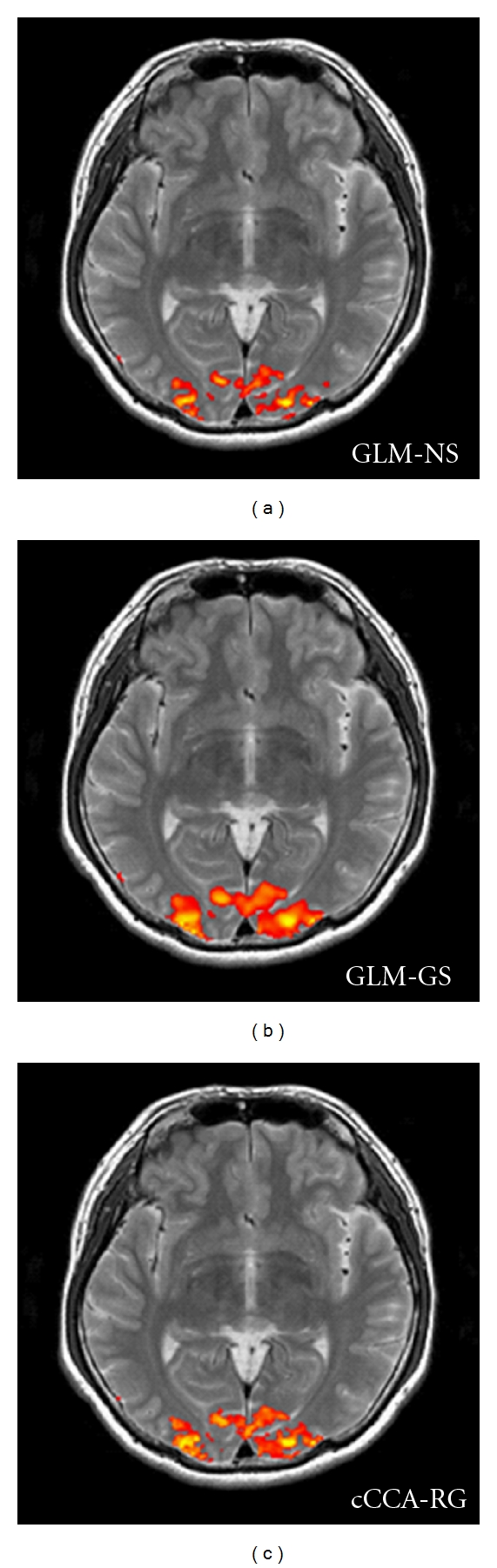
Activation maps for the contrast V-F of the visual paradigm using corrected *P*-values (*P* < 0.05). The GLM-GS method yields the smoothest activation map at the expense of showing activations reaching outside of gray matter. The GLM-NS method preserves activations in gray matter much better but with unappealing broken links among activated voxels. The activation map using cCCA-RG provides a compromise between the smooth appearance of activations and preservation of fine cortical structure.

In the following, we show the activation maps with corrected *P* < 0.05 that are overlaid on their corresponding T2 images. Images in the figures are in radiological convention (left is right and vice versa). We only show them in 2D slices because the current application of cCCA-RG was in 2D, so was GLM-GS for a fair comparison and the (coregistered) activation maps were laid on each individual co-planar T2 image. In Figure 7, we show the activation maps of the contrast V-F of visual data for different methods from one representative subject. It can be seen that GLM-GS yields the smoothest activation map at the expense of loss of the visual cortex structures and GLM-NS preserves these folded structures much better but with some unappealing broken links. The activation map of cCCA-RG provides a good compromise between the smoothness of activations and preservation of fine cortical structure.

**Figure 8:**
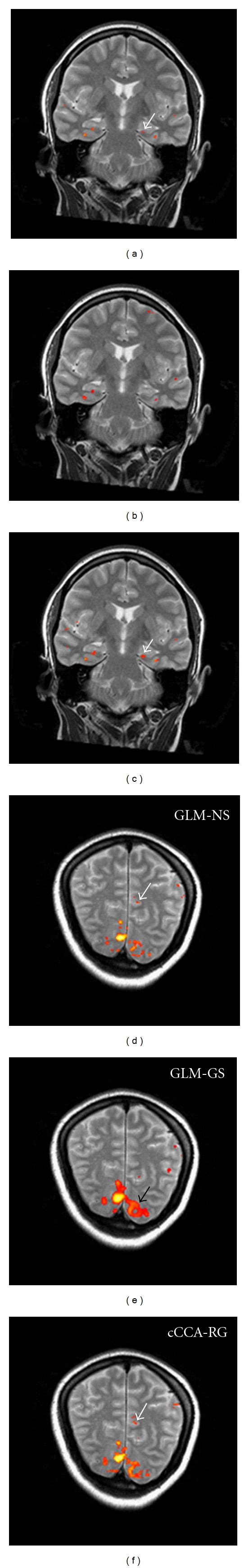
Activation maps for the contrast E-C of the memory paradigm using corrected *P*-values (*P* < 0.05). Upper row: activations in the anterior portion of the hippocampal complex; lower row: activations in the posterior and middle cingulate cortex and in the precuneus. Note that GLM-NS and cCCA-RG lead to symmetric (left and right) activation patterns in the hippocampus and parahippocampal gyrus and also to weak and localized activations in the posterior/middle cingulate cortex (see white arrows). Using GLM-GS, strong activation patterns become overly bulgy (see black arrow). Compared to GLM-NS, cCCA-RG yields more activated voxels and better connected activations confined to gray matter.

The activation maps of the contrast E-C of memory data from another representative subject are shown in Figure 8. The slices shown in the upper row contain an anterior portion of the hippocampal complex. Symmetrical activations in hippocampus and parahippocampal gyrus are detected by GLM-NS and cCCA-RG. The missing activation at the left hippocampus (see white arrows) of GLM-GS demonstrates the undesirable effects of a fixed isotropic Gaussian spatial smoothing on localized weak activation patterns. A more posterior slice is shown in the bottom row. Memory encoding activation is obtained in the posterior cingulate cortex and precuneus. Using the GLM-GS method, activations appear overly bulgy and have some unlikely connections through white matter (shown by the black arrow). Also, small and weak activations in the posterior cingulate cortex (see white arrows) are not shown in the activation map of GLM-GS. The GLM-GS method leads not only to missing activations but also to artifactual activations where a large fraction of false activations show up in white matter and CSF regions due to the spherical (nondirectional) smoothing kernel. The cCCA-RG method yields more activated voxels and better connected activations in gray matter than GLM-NS.

To make a quantitative comparison of the locations of activations of different methods, we used the gray matter mask from the acquired IR-SE-EPI scan and calculated the ratio of the number of activated voxels detected in gray matter and the number of activated voxels detected outside of gray matter (listed in Table 2). This ratio reflects the degree of activations confined to gray matter. As expected, GLM-NS has the highest value because of no smoothing involved. Compared to fixed Gaussian smoothing (GLM-GS), cCCA-RG yields higher ratios, which demonstrates that the adaptive smoothing suffers less blurring outside of gray matter than fixed Gaussian spatial smoothing.

## 5. Discussion

Using the newly developed directional test statistic for cCCA of fMRI data, we are able to compare cCCA with traditional GLM methods for a more complicated memory paradigm. The quantitative results from the simulated and pseudoreal data and the qualitative results from real fMRI data clearly demonstrate that the proposed method (directional test with cCCA) outperformed the conventional GLM with and without Gaussian smoothing. This work paves the way for applying CCA methods for testing general linear contrasts in a more complicated fMRI experimental design.

Our comprehensive evaluation study also provides valuable insights for applying smoothing in fMRI data analysis. The pseudoreal data used in this study can be divided into four situations: (1) spatially extended and strong activation (VIS 0.55); (2) spatially extended and weak activation (VIS 0.65); (3) focal and strong activation (MEM 0.55); (4) focal and weak activation (MEM 0.65). As expected, the smoothing does not provide much benefit for detecting strong activations. The Gaussian smoothing is only effective for the second situation—spatially extended and weak activation (Figures 4 and 5) because the smoothing helps little for detection of strong signals and the isotropic smoothing adversely eliminates the small or irregular weak activation patterns. The adaptive smoothing by cCCA always performed best in all four situations and the biggest advantage takes place for the last situation—focal and weak activations (Figures 4–6). For real fMRI data, the Gaussian smoothing can yield a large block of smooth activations, which are appealing to human visual perception. However, there is a risk of overlooking important subtle activations as well as overestimating the extent of strong activations (Figure 7). As can be seen in Figures 7 and 8, the adaptive smoothing by cCCA yields activation maps that are not only visually appealing (smoothness) but also well localized (along the gyri and sulci of gray matter).

The improved detection performance of cCCA is at the expense of computation. If an exhaustive search is used for the optimization of constrained CCA, the number of CCA computations will be equal to the number of possible voxel configurations in the chosen neighborhood. This number is of the order O(2^*N*−1^), where *N* is the number of voxels in the search area [12, 24]. That means 256 CCA computations for a 3 × 3 in-plane neighborhood and 2^26^ for a 3 × 3 × 3 voxel volume. Heuristic search methods, such as the branch-and-bound algorithm [10, 14] and a region-growing algorithm [12], were used to reduce the computational cost and to maintain the detection performance. The current implementation of cCCA-RG in 2D [12] is feasible for routine fMRI data analysis. For the estimation of a 2D slice with 6317 in-brain pixels, using MATLAB on a computer equipped with Intel Core 2 2.4 GHz CPU and 4 GB memory, cCCA-RG takes about 24 seconds. Although it is about 10 times slower than GLM-NS and GLM-GS, a fully 3D brain volume sequence can be processed within 10 minutes. On the other hand, the rapid evolving computer hardware and parallel computing techniques, for example, GPU computing, can dramatically shorten the time for cCCA in future applications.

Besides CCA [8–13, 24], there exist other methods that use adaptive smoothing techniques for fMRI data analysis (e.g., [36–38]). A quite different method is used in [38], where a propagation-separation procedure is applied on contrast and residual images, obtained by the GLM, to achieve adaptive smoothing of the estimated parameters. The final activation detection is based on random field theory [6]. However, the advantage of preserved shape and geometry of the activation areas and increased signal-to-noise ratio was only demonstrated by simulated data and real motor data, thus the effectiveness of this postestimation smoothing on focal weak activations is unclear. Another test statistic similar to canonical correlation, proposed in [36], is defined as a ratio between the energy of signal space and the energy of residuals. Its power relies on the optimal spatial weighting based on different signal spaces. This method is equivalent to conventional CCA. However, the maximum energy ratio, in its current formulation, does not allow for a more general contrast design, as well as a directional test. Moreover, the estimate and inference have to be done for each signal space, which is computationally expensive. Our test statistics is more general and outperforms the GLM with or without Gaussian smoothing. In addition to its improved sensitivity, advantages are that general contrasts can be defined after the estimation and a directional test is readily available. It is worthwhile to note that adaptive smoothing can also be achieved through spatial priors defined in a Bayesian framework (e.g., [39, 40]), which produces posterior probability maps instead of statistical parametric maps as in classical inference. Though Bayesian methods hold some advantages over classical inference, such as capability of inferring an effect size and no need for multiple comparison correction, the specification of the priors and the likelihood functions may have a large impact on the final results and the computation is usually more complex and time consuming. For comparing all these adaptive smoothing methods, a thorough study needs to be conducted to evaluate their performance from detection performance of different types of brain activations to their computational cost.

It is important to keep in mind that the advantage of adaptive smoothing may diminish in conventional group analysis, where isotropic smoothing is necessary to improve correspondence of imperfectly registered homologous areas. Nevertheless, the usefulness of adaptive smoothing will be greatly appreciated for fMRI-aided neurosurgical planning [41] and region-of-interest analysis of localized brain functions [42].

One issue that this paper has not addressed is the temporal correlation of the noise and a possible correction of the test statistic by prewhitening, as usually done in data analysis using the GLM. Based on the Gauss-Markov theorem, the LS solution of the GLM is the MVU estimator when Gaussian white noise assumption is satisfied, otherwise the weighted LS solution (using the inverse of the noise covariance matrix) becomes the BLUE. For cCCA, a BLUE does not exist because the optimization of the spatial constraints leads to a nonlinear model even though the spatial constraints can be linear [24]. Therefore, unbiasedness of constraint CCA by prewhitening is not possible and non-parametric methods need to be used to obtain accurate *P*-values.

The purpose of this research is to develop a simple directional test statistic for cCCA similar to a *t*-statistic. Given that the HRF is modeled perfectly, a *t*-test, as a likelihood ratio test in the univariate case, is the most sensitive test. For block designs, the canonical 2-gamma function is a good choice for the temporal modeling of the BOLD response. However, in event-related designs, more complicated temporal regressors may be useful (such as first and second derivative of the HRF function) to model the delay and dispersion of the BOLD response. In such a scenario, an unsigned test statistic, for example, *F*-statistic, is preferred to test for the evoked regional effects. A test statistic for CCA similar to *F*-statistic can be derived from Wilks' Λ as

(18) $$F\left(v_{\tilde{H}},v_{\tilde{E}}\right)=\frac{1-\Lambda }{\Lambda }\frac{v_{\tilde{E}}}{v_{\tilde{H}}},$$

where $v_{\tilde{H}}$ and $v_{\tilde{E}}$ are the degrees of freedom of the hypothesis matrix and the error matrix, respectively. The delay and dispersion regressors can be included in our proposed CCA method in the same way as for the GLM since the temporal modeling of the HRF response is the same for both methods.

## 6. Conclusions

In this paper, we derived a novel directional test statistic for CCA so that CCA can handle general linear contrasts in more complicated fMRI paradigms. Using this novel test statistic, different contrasts can be tested after model fitting without reparameterization of the design matrix and reestimating each individual contrast of interest. With the proper constraints on the spatial coefficients of CCA, this CCA statistic can yield a more powerful test than the traditional *t*-test in the GLM, especially for weakly evoked and localized brain activations. This behavior was demonstrated not only by superior performance using simulations and traditional ROC techniques but also by activation maps of real fMRI applications. Since the trend in fMRI is to move toward high-resolution imaging where the signal is weak, the spatial correlation is strong, and the amount of data is enormous, we envision that our method with improved detection power and computation time will be important for future fMRI data analysis.

## Figures and Tables

**Table 1:** Mean square errors (MSEs) of estimated coefficients for different methods. To define different spatial patterns of activations, 100000 randomly shaped activations within a 3 × 3 grid of pixels having a size of 2 to 9 pixels were generated. The corresponding time courses for the activated voxels were simulated to be linear combinations of the 4 random temporal regressors with random amplitudes. Different levels of noise were introduced by resampling 3 × 3 patches of resting-state fMRI data. The mean square errors between the originally simulated amplitudes of regressors and estimated ones are shown. The cCCA-RG method achieves more than 25% less MSE compared to GLM-NS. The GLM-GS method is worse than GLM-NS due to the small and irregularly defined activation patterns.

	Δ*β*_1_^2^	Δ*β*_2_^2^	Δ*β*_3_^2^	Δ*β*_4_^2^
GLM-NS	0.1331	0.1837	0.2200	0.1488
GLM-GS	0.1627	0.2047	0.2336	0.1769
cCCA-RG	0.0979	0.1339	0.1538	0.1091

**Table 2:** Ratio of activations in gray matter and outside of gray matter for different analysis methods. The number in the table is the ratio of the number of voxels detected in gray matter and the number of voxels detected outside of gray matter. Note that the GLM-NS has the highest value because there is no smoothing involved. Compared to fixed Gaussian spatial smoothing (GLM-GS), cCCA-RG yields higher ratios demonstrating less blurring and better confinement to gray matter.

	GLM-NS	GLM-GS	cCCA-RG
V−F	6.21	2.84	3.35
E−C	1.81	1.32	1.53
